# Anxiety and depression in patients with non-site-specific cancer symptoms: data from a rapid diagnostic clinic

**DOI:** 10.3389/fonc.2024.1358888

**Published:** 2024-06-03

**Authors:** Maria J. Monroy-Iglesias, Beth Russell, Sabine Martin, Louis Fox, Charlotte Moss, Flaminia Bruno, Juliet Millwaters, Lindsay Steward, Colette Murtagh, Carlos Cargaleiro, Darren Bater, Grace Lavelle, Anna Simpson, Jemima Onih, Anna Haire, Clare Reeder, Geraint Jones, Sue Smith, Aida Santaolalla, Mieke Van Hemelrijck, Saoirse Dolly

**Affiliations:** ^1^ Translational Oncology and Urology Research, King’s College London, Faculty of Life Sciences and Medicine, London, United Kingdom; ^2^ Medical Oncology, Guy’s and St Thomas’ National Health System (NHS) Foundation Trust, London, United Kingdom; ^3^ King’s College London, Institute of Psychiatry, Psychology and Neuroscience, Department of Psychological Medicine, London, United Kingdom; ^4^ Macmillan Psychological Support (MAPS) Team, Lewisham and Greenwich NHS Trust, London, United Kingdom; ^5^ South East London Cancer Alliance, London, United Kingdom; ^6^ Dimbleby Cancer Care, Guy’s Cancer Centre, Guy’s and St Thomas’ National Health System (NHS) Foundation Trust, London, United Kingdom

**Keywords:** anxiety, depression, non-site specific, cancer, machine learning, standard statistics

## Abstract

**Background:**

Rapid diagnostic clinics (RDCs) provide a streamlined holistic pathway for patients presenting with non-site specific (NSS) symptoms concerning of malignancy. The current study aimed to: 1) assess the prevalence of anxiety and depression, and 2) identify a combination of patient characteristics and symptoms associated with severe anxiety and depression at Guy’s and St Thomas’ Foundation Trust (GSTT) RDC in Southeast London. Additionally, we compared standard statistical methods with machine learning algorithms for predicting severe anxiety and depression.

**Methods:**

Patients seen at GSTT RDC between June 2019 and January 2023 completed the General Anxiety Disorder Questionnaire (GAD-7) and Patient Health Questionnaire (PHQ-8) questionnaires, at baseline. We used logistic regression (LR) and 2 machine learning (ML) algorithms (random forest (RF), support vector machine (SVM)) to predict risk of severe anxiety and severe depression. The models were constructed using a set of sociodemographic and clinical variables.

**Results:**

A total of 1734 patients completed GAD-7 and PHQ-8 questionnaires. Of these, the mean age was 59 years (Standard Deviation: 15.5), and 61.5% (n:1067) were female. Prevalence of severe anxiety (GAD-7 score ≥15) was 13.8% and severe depression (PHQ-8 score≥20) was 9.3%. LR showed that a combination of previous mental health condition (PMH, Adjusted Odds Rario (AOR) 3.28; 95% confidence interval (CI) 2.36–4.56), symptom duration >6 months (AOR 2.20; 95%CI 1.28–3.77), weight loss (AOR 1.88; 95% CI 1.36–2.61), progressive pain (AOR 1.71; 95%CI 1.26–2.32), and fatigue (AOR 1.36; 95%CI 1.01–1.84), was positively associated with severe anxiety. Likewise, a combination PMH condition (AOR 3.95; 95%CI 2.17–5.75), fatigue (AOR 2.11; 95%CI 1.47–3.01), symptom duration >6 months (AOR 1.98; 95%CI 1.06–3.68), weight loss (AOR 1.66; 95%CI 1.13–2.44), and progressive pain (AOR 1.50; 95%CI 1.04–2.16), was positively associated with severe depression. LR and SVM had highest accuracy levels for severe anxiety (LR: 86%, SVM: 85%) and severe depression (SVM: 89%, LR: 86%).

**Conclusion:**

High prevalence of severe anxiety and severe depression was found. PMH, fatigue, weight loss, progressive pain, and symptoms >6 months emerged as combined risk factors for both these psychological comorbidities. RDCs offer an opportunity to alleviate distress in patients with concerning symptoms by expediting diagnostic evaluations.

## 1 Introduction

Undergoing a diagnostic evaluation for suspected cancer can be perceived as stressful and may cause psychological distress, because of the threat of being seriously ill, the uncertainty of unexplained symptoms, or the invasiveness of various cancer investigations (1). Prior studies analysing the link between cancer investigations, and psychological distress, which commonly include anxiety, low mood, stress, worry, panic, and fear, have been inconsistent. Various studies have linked cancer investigations with a high prevalence of distress (2–5). However, two recent systematic reviews reported a low prevalence of psychological distress across different types of cancer, except for colorectal cancer (CRC) screening due to the invasiveness of the procedures (e.g., endoscopies) as part of their diagnostic work-up (6, 7).

In the context of patients who present with non-site specific (NSS) symptoms concerning of cancer, fewer studies have investigated levels of psychological distress. To expedite cancer and serious benign diagnoses and improve patient’s experience in those with concerning NSS symptoms (e.g., weight loss, fatigue, vague abdominal pain) (8), rapid diagnostic clinics (RDCs) have been established. RDCs aim to provide a NSS pathway by prioritising diagnostics, whilst prehabilitating patients by managing comorbidities, polypharmacy, nutrition, and mental health conditions (9). To our knowledge, only one study has investigated psychological distress in those presenting with NSS symptoms in an RDC setting (1). This Danish study reported on quality of life (QoL) scores of patients with NSS symptoms and noted that those suspected of cancer had similar QoL scores (including anxiety and depression) at the outset of their diagnostic evaluation compared to after their evaluation, irrespective of their final diagnosis. In addition, those individuals who ultimately did not receive a cancer diagnosis experienced an improvement in their QoL scores and a reduction in their symptom burden (1). Furthermore, NSS symptoms may also be indicative of an underlying generalized anxiety or major depressive disorder. Diagnostic criteria outlined in the Diagnostic and Statistical Manual of Mental Disorders, fifth edition (DSM-5), include significant weight loss, decrease of appetite, fatigue, and sleep disturbances for major depressive disorder and fatigue, sleep disturbances, and muscle tension for generalized anxiety disorder (10, 11). Therefore, it is crucial to differentiate whether NSS symptoms stem from a pre-existing mental health condition or an underlying organic cause, so that both the physical and psychological needs of these patients can be properly managed.

Given the limited knowledge about psychological distress in the NSS population we used data from the RDC at Guy’s and St Thomas’ Foundation Trust (GSTT), which was established in 2016 and has seen over 5,000 patients to date to (1): assess the prevalence of anxiety and depression; (2) to identify patterns of patient characteristics and symptoms information that can be associated with anxiety and depression at baseline in this specific patient population. Additionally, we aimed to compare the model performance of a traditional statistical method, with machine learning algorithms in predicting outcomes within our population.

## 2 Methods

### 2.1 Study setting

GSTT RDC was established to provide an efficient pathway for adult patients (i.e., 18 years and over) in southeast London experiencing vague symptoms suggestive of cancer, who do not meet site-specific urgent cancer 2-week-wait criteria or fall into multiple diagnostic pathways (12). During their initial consultation, patients undergo a comprehensive assessment, including an evaluation of symptoms, physical comorbidities, polypharmacy, lifestyle, social factors, mental health, and nutrition. GSTT RDC employs a holistic, multi-disciplinary approach with specialized consultants in oncology, acute medicine, and general practice. Diagnostic tests, such as blood work, imaging, and endoscopy, are conducted within two weeks of the initial consultation. The goal of GSTT RDC is to expedite diagnosis, refer patients for further evaluation, or discharge them within a 28-day timeframe. A more detailed description of GSTT RDC was published by Dolly et al. (8).

### 2.2 Study population and data collection

**Figure 1:**
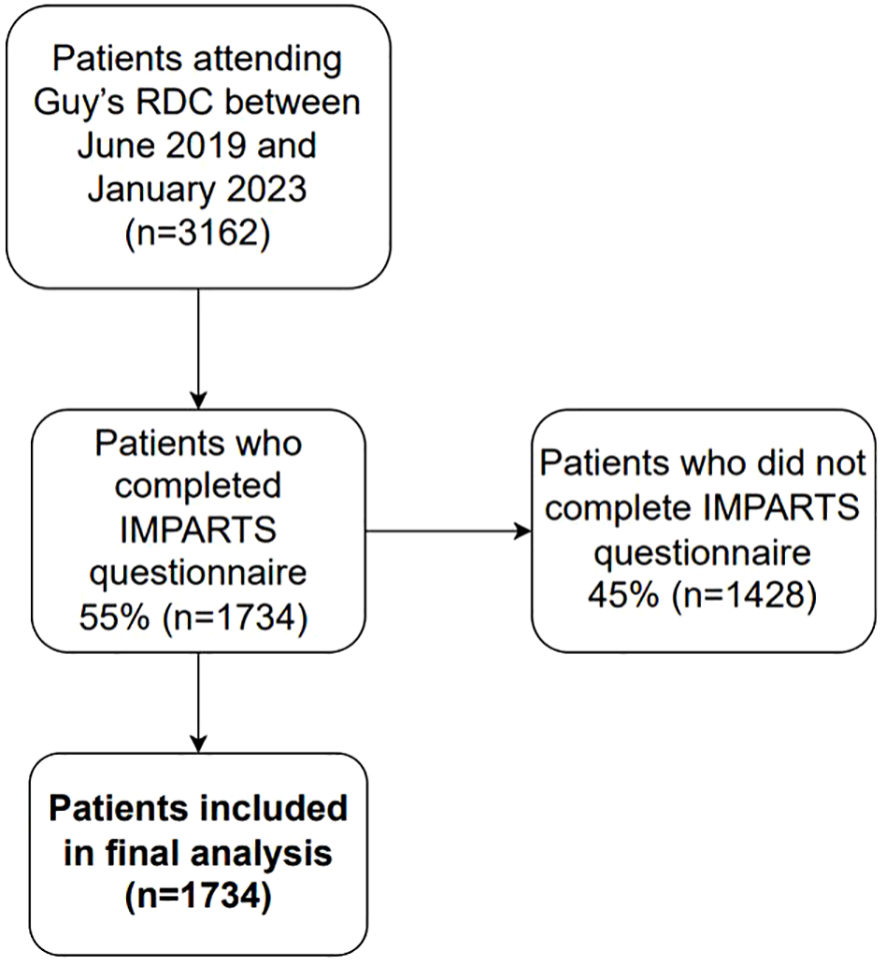
Study population.

Our cohort study included both prospective and retrospective data from patients attending GSTT RDC between June 2019 and January 2023. Data on anxiety and depression were collected during the initial RDC clinic visit as part of the Integrating Mental and Physical Healthcare: Research, Training and Services (IMPARTS) program (13). IMPARTS is a service development platform designed to routinely collect patient-reported outcomes relating to various aspects of mental and physical health, including the General Anxiety Disorder (GAD7) and Patient Health Questionnaire (PHQ8) scales (11). IMPARTS questionnaires were sent electronically to patients one day prior to their first RDC consultation. Patients who did not fill out the questionnaire beforehand were offered to do so electronically on the day of their first consultation with help from a healthcare assistant. After completion, patient responses were uploaded to their electronic health records and integrated into an online data collection tool, accessible solely to IMPARTS staff (G.L. and J.O.), who subsequently provided the data to the research team (MJMI) for analysis. Patients who did not fill out an IMPARTS questionnaire were excluded from subsequent analyses (45%, n=1428; **Figure 1**), resulting in a final study population of 1734 patients. **Supplementary Table 1** summarizes the clinical and sociodemographic characteristics of questionnaire completers compared to non-completers.

Clinical and sociodemographic data were collected retrospectively from the electronic patient records (EPR) for all patients seen between June 2019 and May 2020. For patients seen from June until 2020 onwards, data were collected prospectively and managed using REDCap electronic data capture tools hosted at GSTT (14). Non-patient reported data, such as sex at birth, age at first RDC consultation, ethnicity, socioeconomic status (based on the Index of Multiple Deprivation Score (IMD) (15)), smoking status, comorbidities, previous mental health (MH) condition, performance status (0–4, indicating patients’ level of functioning (16)), symptom duration, and presenting symptoms (weight loss, fatigue, vague abdominal pain, progressive pain) were collected.

Lastly, patients who opted out of the NHS Digital National data opt out programme were excluded from analysis (n=382) (17). All data, including IMPARTS questionnaire information, were collected and analysed under the ethical approval of GSTT Cancer Cohort (Ethics reference number: 23/NW/0105) and Health Research Authority South Central – Oxford C (Ethics reference number: 23/SC/0109).

### 2.3 Anxiety and depression assessment

The 7-item GAD7 scale was used for measuring anxiety, while the data on depression was collected using the 8-item PHQ8 scale. The GAD7 score was estimated by assigning the scores of 0, 1, 2, and 3 to the response categories of “not at all”, “several days”, “more than half the days”, and “nearly every day”, respectively for questions related to anxiety. The scores of 5, 10, and 15 were taken as cutoff points for mild, moderate, and severe anxiety (18). The PHQ8 items are composed of the same response categories as GAD7, for questions related to depression. The scores of 5, 10, 15, and 20 were taken as cutoff points for mild, moderate, moderately severe, and severe, respectively (19). Both GAD7 and PHQ8 were converted into binary variables, where abnormal values were classified as severe anxiety (GAD7 score ≥15) and severe depression (PHQ8 score ≥20). The GAD7 and PHQ8 questionnaires have been extensively validated in previous research (20–23), demonstrating high levels of validity and reliability.

### 2.4 Data analysis

#### 2.4.1 Missing data

Multiple imputation with chained equations was used to impute data in variables with 9–22% of missing data (i.e., weight loss, fatigue, progressive pain, previous MH condition, performance status, symptom duration, comorbidity number, and ethnicity) under the missing at random assumption. The imputation contained all other candidate predictor variables and the endpoint indicators (i.e., severe anxiety, severe depression). We generated ten imputations and used these in all model fitting and evaluation steps. After model fitting was performed in each imputed dataset, the results were pooled using Rubin’s rules.

#### 2.4.2 Logistic regression

Logistic regression models were used to calculate odds ratios (OR) and 95% confidence intervals (95%CI) for risk of severe anxiety and severe depression. To mitigate potential confounding effects of physical symptoms on anxiety and depression scores, particularly in patients presenting with NSS symptoms, we focused our analysis on severe levels of anxiety and depression as outcomes. This approach helped minimize the risk of including inflated scores that may be influenced by physical symptoms, ensuring a more accurate representation of true psychological distress.

Furthermore, the following patient characteristics and symptoms were considered in the models: sex at birth (male, female), age (<25, 25–50, 50–75, >75 years), ethnicity (White, Black, Other), socioeconomic status (low (IMD score of ≤3), middle (4–7), high (≥8)), smoking status (never, ever, current), comorbidities (0, 1, 2+), previous MH condition (yes, no), performance status (0, 1, 2, 3, 4), symptom duration (<1 month, 1–3 months, 3–6 months, >6 months), weight loss (yes, no), fatigue (yes, no), vague abdominal pain (yes, no), and progressive pain (yes, no). All variables were mutually adjusted for each other in the analysis. The goodness-of-fit of the logistic regression model was evaluated using the Hosmer-Lemeshow test.

Moreover, to assess multicollinearity among the variables included in full logistic regression model, we applied stepwise regression to fit the model. This method gradually fitted the model by removing covariates with a p-value greater than 0.1 and adding covariates with a p-value less than 0.05.

#### 2.4.3 Machine learning algorithms

To complement standard regression models, we built two machine learning (ML) predictive models (random forest (RF) and support vector machine (SVM)). The data was divided into a 70:30 ratio, representing training and testing, respectively. The cross-validation approach with grid search method was used for parameter optimization. The parameters for both algorithms were explored, and the optimal settings were determined. For the RF model, after experimentation with 50 and 100 trees, the final configuration comprised 75 trees, which yielded the highest accuracy. The SVM regularization parameter was set to 20, and the radial basis function kernel was set to 0.001.

#### 2.4.4 Performance evaluation of regression models and ML models

Different performance measures were used to evaluate whether the models can predict severe depression and anxiety, such as accuracy, specificity, positive predictive value (PPV, precision), sensitivity (recall), and F1-score. The equations below were used to calculate the performance parameters based on confusion matrices (24).

Specificity = True Negative / (False Positive + True Negative)

PPV (Precision) = True Positive / (True Positive + False Positive)

Sensitivity (Recall) = True Positive / (True Positive + False Negative)

F-measure = (2 × Precision × Recall) / (Precision + Recall)

Accuracy = (True Positive + True Negative) / (True Positive + True Negative + False Positive + False Negative)

Error rate = 1- Accuracy

Data processing, multiple imputation, regression modelling and machine learning modelling were performed in R 4.0.1 (glmnet, randomForest, e1071 packages), and Stata 18 (Stata Corporation, College Station, TX).

## 3 Results

A total of 3,161 patients attended GSTT RDC between June 2019 and January 2023. Of these, 1,734 (55%) patients completed an IMPARTS questionnaire and were therefore included in the study. **Supplementary Table 1** summarizes the patient characteristics of those who completed the IMPARTS questionnaire compared to those who did not. Both groups exhibited similar characteristics, except for age groups, whereby older patients (≥75 years) were underrepresented amongst questionnaire completers (40.3% completion rate) compared with younger subgroups (57%-63% completion rates. The demographic characteristics of the patients who completed an IMPARTS questionnaire are summarized in **Table 1**. The patient cohort comprised 38.5% (n=667) males, with an average age of 59 years (SD 15.5 years). The distribution of age groups was: 1.7% (n=30) <25 years, 25.6% (n=443) 25–50, 57.6% (n=998) 50–75, and 15.2% (n=263) 75+ years. Ethnicity-wise, 53.2% (n=922) identified as White, 19.3% (n=334) as Black, and 11.5% (n=200) represented other ethnicities. The majority (63.9%, n=1108) of patients had two or more comorbidities, but in terms of performance status (PS), 55.4% (n=960) scored 0. Interestingly, 15.1% (n=262) of participants had a previously identified mental health condition.

**Table 1:** Characteristics of final study cohort.

	Study Population n=1734
Sex	
**Male**	667 (38.5%)
**Female**	1067 (61.5%)
Age at diagnosis	
**Mean (SD)**	59 (15.5)
**Median (IQR)**	60 (49–71)
**<25**	30 (1.7%)
**25–50**	443 (25.6%)
**50–75**	998 (57.6%)
**>75**	263 (15.2%)
Index of Multiple Deprivation	
**Mean (SD)**	4.9 (2.5)
**Low income (1–3)**	640 (36.9%)
**Middle income (4–7)**	764 (44.1%)
**High income (8–10)**	330 (19%)
Ethnicity	
**White**	922 (53.2%)
**Black**	334 (19.3%)
**Other**	200 (11.5%)
**Not known**	278 (16%)
Smoking status	
**Never**	778 (44.9%)
**Current**	404 (23.3%)
**Former**	313 (18.1%)
**Not known**	239 (13.8%)
Comorbidities	
**0**	57 (3.3%)
**1**	202 (11.7%)
**2+**	1108 (63.9%)
**Not known**	367 (21.2%)
Previous mental health illness	
**Yes**	262 (15.1%)
**No**	1224 (70.6%)
**Not known**	248 (14.3%)
Performance status	
**0**	960 (55.4%)
**1–2**	492 (28.4%)
**3–4**	41 (2.4%)
**Not known**	241 (13.8%)
Symptom duration	
**<1 month**	157 (9.1%)
**1–3 months**	261 (15.1%)
**3–6 months**	384 (22.2%)
**>6 months**	600 (34.6%)
**Not known**	332 (19.2%)
Weight loss	
**Yes**	909 (52.4%)
**No**	655 (37.8%)
**Not known**	170 (9.8%)
Fatigue	
**Yes**	565 (32.6%)
**No**	988 (57%)
**Not known**	181 (10.4%)
Vague abdominal pain	
**Yes**	496 (28.6%)
**No**	1068 (61.6%)
**Not known**	170 (9.8%)
Progressive pain	
**Yes**	567 (32.7%)
**No**	997 (57.5%)
**Not known**	170 (9.8%)
Primary diagnosis	
**Cancer**	84 (4.8%)
**Serious benign condition**	115 (6.6%)
**Non serious benign condition**	551 (31.8%)
**None/Other**	984 (56.8%)

**Table 2** demonstrates the prevalence of anxiety and depression, based on GAD-7 and PHQ-8 scores, within our study population. Among participants, 8.9% had mild anxiety (95%CI 7%-10%), 12.1% had moderate anxiety (95%CI 10%-13%), and 13.8% experienced severe anxiety (95%CI 12%-15%). Additionally, 7.4% reported mild depression (95%CI 6%-8%), 14.2% had moderate depression (95%CI 12%-15%), 11.9% reported moderately severe depression (95%CI 10%-13%), and 9.3% indicated severe depression (95%CI 7%-10%). Lastly, 6.4% (n=111) of patients had both severe anxiety and severe depression.

**Table 2:** Prevalence of anxiety and depression within our study population.

	Study Population n=1734
Anxiety (GAD-7) scores	
**None (≤4)**	1131 (65.2%; 95%CI 62%-67%-)
**Mild (5–9)**	154 (8.9%; 95%CI 7%-10%)
**Moderate (10–14)**	209 (12.1%; 95%CI 10%-13%)
**Severe (≥15)**	240 (13.8%; 95%CI 12%-15%)
Depression (PHQ-8) scores	
**None (≤4)**	990 (57.1%; 95%CI 54%-59%)
**Mild (5–9)**	129 (7.4%; 95%CI 6%-8%)
**Moderate (10–14)**	247 (14.2%; 95%CI 12%-15%)
**Moderately severe (15–19)**	206 (11.9%; 95%CI 10%-13%)
**Severe (≥20)**	162 (9.3%; 95%CI 7%-10%)
**Severe anxiety (GAD7 ≥15 and severe depression (PHQ-8 ≥20)**	111 (6.4%; 95%CI 5%-7%)

CI, Confidence Interval.

### 3.1 Missing data

Missing data for variables in our analysis varied, with percentages as follows: 9.8% for weight loss, 10.4% for fatigue, 9.8% for progressive pain, 14.3% for previous MH condition, 13.8% for smoking status, 13.9% for performance status, 19.2% for symptom duration, 21.2% for comorbidity number, and 16% for ethnicity.

### 3.2 Logistic regression

#### 3.2.1 Anxiety

Results of multivariable logistic regression for risk of severe anxiety are shown in **Table 3**. Model performance is summarized in **Table 4**. In terms of demographic factors, no statistically significant associations were observed for age, socioeconomic status, or ethnicity. However, weight loss, fatigue, progressive pain, symptom duration, and previous mental health condition did show an association with risk of severe anxiety. For example, patients who reported weight loss had significantly increased odds of severe anxiety (adjusted OR of 1.94 (95% CI 1.40–2.68)), similar to those experiencing progressive pain (adjusted OR of 1.69 (95% CI 1.24–2.30)). The Hosmer-Lemeshow test for goodness-of-fit yielded a non-significant result (Chi-square = 897.49, df = 897, p = 0.4891), indicating that the logistic regression model for severe anxiety adequately fits the data.

**Table 3:** Odds ratios (OR) and 95% confidence intervals (CI) for sociodemographic and clinical characteristics by risk of severe anxiety and depression.

		Severe Anxiety				Severe Depression			
		Severe Anxiety n (%)	No Severe Anxiety n (%)	OR*	95% CI	Severe Depression n (%)	No Severe Depression n (%)	OR*	95% CI
Sociodemographic characteristics									
Sex	Male	587 (39.3)	80 (33.3)	1.00	Ref	618 (39.3)	49 (30.3)	1.00	Ref
	Female	907 (60.7)	160 (66.7)	1.19	0.86–1.63	954 (60.7)	113 (69.7)	1.32	0.90–1.94
Age	<25 years	27 (1.8)	3 (1.3)	1.00	Ref	20 (1.8)	2 (1.2)	1.00	Ref
	25–50 years	387 (25.9)	56 (23.3)	1.34	0.37–4.89	402 (25.6)	41 (25.3)	1.62	0.35–7.56
	50–75 years	854 (57.2)	144 (60)	1.37	0.41–5.31	901 (57.3)	97 (59.9)	1.66	0.36–7.63
	>75 years	226 (15.1)	37 (15.4)	1.65	0.44–6.23	241 (15.3)	22 (13.6)	1.74	0.35–8.49
Income (IMD)**	Low	525 (35.1)	115 (47.9)	1.00	Ref	560 (35.6)	80 (49.4)	1.00	Ref
	*Middle*	*668 (44.7)*	*96 (40)*	*0.67*	*0.48–0.92*	*703 (44.7)*	*61 (37.6)*	*0.59*	*0.41–0.87*
	*High*	*301 (20.2)*	*29 (12.1)*	*0.44*	*0.27–0.71*	*309 (19.7)*	*21 (13)*	*0.45*	*0.25–0.77*
Ethnicity	White	797 (64)	125 (59.5)	1.00	Ref	560 (35.6)	80 (49.4)	1.00	Ref
	Black	283 (22.7)	51 (24.3)	0.94	0.65–1.36	703 (44.7)	61 (37.6)	0.84	0.54–1.32
	Other	166 (13.3)	34 (16.2)	1.28	0.83–1.97	309 (19.7)	21 (13)	1.14	0.68–1.90
Smoking status	Never	710 (52.3)	68 (49.3)	1.00	Ref	675 (52.4)	103 (49.8)	1.00	Ref
	Current	376 (27.7)	28 (20.3)	0.93	0.64–1.35	357 (27.7)	47 (22.7)	0.86	0.54–1.35
	Former	271 (20)	42 (30.4)	1.08	0.74–1.58	256 (19.9)	57 (27.5)	1.13	0.73–1.74
Presenting symptoms									
Weight loss**	No	591 (44)	64 (29)	1.00	Ref	611 (43.2)	44 (29.7)	1.00	Ref
	*Yes*	*753 (56)*	*156 (71)*	*1.88*	*1.36–2.61*	*805 (56.8)*	*104 (70.3)*	*1.66*	*1.13–2.44*
Fatigue**	No	870 (65.2)	118 (53.9)	1.00	Ref	924 (65.7)	64 (43.8)	1.00	Ref
	*Yes*	*464 (34.8)*	*101 (46.1)*	*1.36*	*1.01–1.84*	*483 (34.3)*	*82 (56.2)*	*2.11*	*1.47–3.01*
Abdominal pain	No	929 (69.1)	139 (63.2)	1.00	Ref	975 (68.9)	93 (62.8)	1.00	Ref
	Yes	415 (30.9)	81 (36.8)	1.12	0.82–1.53	441 (31.1)	55 (37.2)	0.98	0.68–1.90
Progressive Pain**	No	887 (66)	110 (50)	1.00	Ref	923 (65.2)	74 (50)	1.00	Ref
	*Yes*	*457 (34)*	*110 (50)*	*1.71*	*1.26–2.32*	*493 (34.8)*	*74 (50)*	*1.50*	*1.04–2.16*
Symptom duration**	<1 month	141 (11.7)	16 (8)	1.00	Ref	145 (11.4)	12 (9.2)	1.00	Ref
	1–3 months	224 (18.6)	37 (18.6)	1.76	0.98–3.18	242 (19)	19 (14.5)	1.25	0.62–2.53
	3–6 months	335 (27.9)	49 (24.7)	1.64	0.93–2.90	348 (27.4)	36 (27.5)	1.69	0.88–3.25
	>6 months	*503 (41.8)*	*97 (48.7)*	*2.20*	*1.28–3.77*	*536 (42.2)*	*64 (48.8)*	*1.98*	*1.06–3.68*
Clinical characteristics									
Previous MH condition**	No	1,095 (85.5)	129 (62.6)	1.00	Ref	1,149 (85)	75 (56)	1.00	Ref
	*Yes*	*185 (14.5)*	*77 (37.4)*	*3.28*	*2.36–4.56*	*203 (15)*	*59 (44)*	*3.95*	*2.71–5.75*
Number of comorbidities	0	51 (4.3)	6 (3.3)	1.00	Ref	54 (4.3)	3 (2.5)	1.00	Ref
	1	188 (15.8)	14 (7.8)	0.64	0.26–1.55	192 (15.5)	10 (8.1)	0.84	0.28–2.55
	2+	948 (19.9)	160 (88.9)	0.88	0.40–1.92	998 (80.2)	110 (89.4)	1.15	0.42–3.11
Performance Status	0	855 (66.3)	105 (51.5)	1.00	Ref	888 (65.6)	72 (51.8)	1.00	Ref
	1	329 (25.5)	62 (30.4)	1.14	0.81–1.61	346 (25.6)	45 (32.4)	1.12	0.75–1.67
	2	75 (5.8)	26 (12.8)	1.92	1.16–3.16	85 (6.3)	16 (11.5)	1.44	0.78–2.64
	3	20 (1.5)	7 (3.4)	2.39	1.00–5.71	23 (1.7)	4 (2.9)	1.72	0.59–4.99
	4	10 (0.9)	4 (2)	2.73	0.81–9.15	12 (0.8)	2 (1.4)	1.82	0.38–8.55

OR, odds ratio; CI, confidence interval; IMD, Index of Multiple Deprivation; MH, mental health.

*All covariates were adjusted for each other.

**Variables with statistically significant odds ratios include income, weight loss, fatigue, progressive pain, symptom duration, and previous mental health condition.

**Table 4:** Performance measure analysis for different methods used.

		AUC	Accuracy	Error rate	Precision	Recall	Specificity	F1 Score
**Logistic Regression**	**Anxiety**	0.75	0.86	0.13	0.46	0.06	0.98	0.11
	**Depression**	0.78	0.86	0.09	0.42	0.04	0.99	0.07
**Random Forest**	**Anxiety**	0.49	0.82	0.17	0.12	0.03	0.96	0.05
	**Depression**	0.50	0.84	0.15	0.24	0.03	0.98	0.05
**SVM**	**Anxiety**	0.50	0.85	0.15	0.23	0.04	0.95	0.05
	**Depression**	0.57	0.89	0.10	0.26	0.05	0.97	0.06

Furthermore, we conducted stepwise regression to refine our model, which identified six variables—socioeconomic status, weight loss, fatigue, progressive pain, symptom duration, and previous mental health condition—as having strong predictive power. Notably, these variables, except for socioeconomic status, were also identified as significant in our full logistic regression model, demonstrating consistent odds ratios. The detailed results of the stepwise regression model can be found in **Supplementary Table 2.**

#### 3.2.2 Depression

Results of multivariable logistic regression for risk of severe depression are shown in **Table 3**. Model performance is summarized in **Table 4**. In terms of demographic factors, no statistically significant associations were identified for age or ethnicity. However, socioeconomic status, weight loss, fatigue, progressive pain, symptom duration, and a previous mental health condition were all found to be associated with increased odds of severe depression. For instance, patients in middle- and high-income groups exhibited decreased odds of severe depression (OR 0.59; 95% CI 0.40–0.87 and OR 0.45; 95% CI 0.25–0.77, respectively) compared to those with low income. Patients reporting weight loss had increased odds of severe depression (adjusted OR of 1.66 (95% CI 1.13–2.44)), and those experiencing fatigue had substantially higher odds (adjusted OR of 2.11 (95% CI 1.47–3.01)). The Hosmer-Lemeshow test for goodness-of-fit yielded a non-significant result (Chi-square = 858.90, df = 897, p = 0.8151), indicating that the logistic regression model for severe depression adequately fits the data.

Furthermore, the variables identified through stepwise regression for depression risk, including socioeconomic status, weight loss, fatigue, progressive pain, symptom duration, and a previous mental health condition, demonstrated consistent odds ratios with the full logistic regression model. The detailed results of the stepwise regression model for depression can be found in **Supplementary Table 2.**

### 3.3 Machine learning algorithms

**Table 2** demonstrates the comparison between the full logistic regression, and ML algorithms’ accuracy rates for the models used. Logistic regression had the highest accuracy rate for anxiety (86%), while SVM had the highest accuracy rate for depression (89%). Further performance metrics for all models are shown in **Table 3** (area under the curve (AUC), accuracy, error rate, F1-score, PPV, specificity, and sensitivity). The features importance ranking for depression and anxiety were analysed using the random forest (RF) ranking method. The most important features were the same for both severe anxiety and severe depression: number of comorbidities, symptom duration, performance status, ethnicity, and age (**Supplementary Table 3**).

## 4 Discussion

In our study of 1,734 patients attending GSTT RDC we found a prevalence of 13.7% for severe anxiety, and 9.3% for severe depression. The combination of the following patient characteristics and symptoms was positively associated with both risk of severe anxiety and severe depression in patients presenting with NSS symptoms in the context of cancer: lower income, weight loss, fatigue, progressive pain, symptom duration exceeding one month, and a previous mental health condition.

As of 2023, the UK Office for National Statistics (ONS) reported a self-reported anxiety rate of 26.6% in women and 20% in men (25). Remarkably, these rates align closely with those observed within our NSS population, where the prevalence of moderate to severe anxiety was 25.9%. Moreover, a recent study focusing on the prevalence of depression, as measured by PHQ-8 scores, in the broader UK population revealed a severe depression rate of 3.3% (26). In contrast, our study identified a nearly threefold higher prevalence of severe depression at 9.3% within the NSS population. This difference may be attributed to the PHQ-8 questions, such as those related to feeling tired or having little energy, potentially inflating scores. This wording could encompass both mental health patients and those with somatic complaints, warranting further examination of the tool’s impact on the observed differences in depression prevalence within our NSS population. Furthermore, when looking at other NSS populations, only one previous study has looked at psychological distress and other QoL measures in patients with NSS symptoms prior to diagnosis and 30 days after referral, a point in time when diagnostics should have been completed (1). In this study, patients undergoing diagnostic evaluations for cancer via the Cancer Patient Pathway for Serious Non-Specific Symptoms and Signs of Cancer (NSSC-CPP) in Denmark exhibited a high prevalence of mild to severe anxiety (~35%) and depression (~25%) before diagnosis. Notably, patients without a cancer diagnosis experienced a substantial improvement in QoL measures 30 days after referral. Our study aligns with these findings, showing that 35% of our patients scored mild to severe anxiety, and 43% scored mild to severe depression at baseline. Further studies are needed looking into post-diagnosis measures of psychological well-being and QoL assessments within our study population.

Furthermore, psychological distress commonly concurs with chronic health conditions, which may negatively impact QoL, and health related outcomes associated with anxiety and depression (27–29). Whilst an RDC is in the first instance designed to speed up cancer diagnostics, only 7% of our patients ultimately have a cancer diagnosis (8). As such, RDCs provide an avenue to triage patients with other comorbidities and ensure support for psychological distress such as anxiety and depression. Fatigue and comorbidity were both identified in the models for anxiety and depression, highlighting the importance of further referrals for these patients – irrespective of a cancer diagnosis. Additionally, RDCs follow the faster diagnostic framework established by the National Health Service in England (NHSE). This framework ensures patients will be diagnosed or have cancer ruled out within 28 days of being referred urgently by their GP (30). Consequently, RDCs offer a promising avenue for swiftly improving QoL in patients distressed by concerning symptoms.

NSS symptoms (e.g., fatigue, chronic pain) have been consistently linked with a high incidence of anxiety and depression (31). In fact, all patient characteristics identified in our models have been independently associated with anxiety and depression in various other studies (32–35). Thus, it is important to note that their combination is indicative of severe presentation of anxiety and depression in NSS symptom patients. Conversely, it is worth considering that underlying anxiety and/or depressive disorders might be contributing to NSS symptoms. For instance, patients with anxiety and/or depression often report higher levels of pain intensity compared to the general population, and the remission of an underlying depression has been linked to reduced pain severity (27, 36). Additionally, as previously mentioned, the DSM-V outlines various NSS symptoms (i.e., weight loss and fatigue), within their criteria for major depressive disorder and generalized anxiety disorder (10, 37). Hence, embedding a psychology specialist and conducting a mental health assessment during diagnostic workup at our centre could prove beneficial for patients presenting with NSS symptoms, in addition to having a direct access to mental health support and other interventions. Moreover, recognizing the impact of a previous mental health condition is paramount. Patients with a history of mental health illness may continue to demonstrate signs of distress, exacerbating their NSS symptoms.

Additionally, the majority of patients presenting with severe anxiety and depression received a final diagnosis of a non-serious benign condition or had no definitive diagnosis. Specifically, 38.5% and 36.4% of patients with severe anxiety and depression, respectively, fell into this category. Furthermore, among cancer patients, only a small proportion—6.7% for severe anxiety and 6.8% for severe depression—were diagnosed with cancer. This observation supports the hypothesis that NSS symptoms may often reflect underlying psychological distress (**Supplementary Table 4**).

Lastly, this study compared the predictive accuracy of various machine learning algorithms with standard logistic regression modelling in forecasting depression and anxiety. The methods effectively predicted both conditions, with SVM achieving the highest accuracy for depression and logistic regression for anxiety. Despite their success, ML methods did not confer additional advantages within our current dataset due to its nature (electronic health records and patient reported data) and size. ML, although capable of handling several variables and complex interactions, requires larger datasets for accurate analysis (24, 38). In addition, the “black box” nature of ML models (i.e., challenging interpretation), raises transparency concerns, even with tools like variable importance within RF. On the other hand, standard statistics offer simpler interpretation of results, assuming a known relationship between input variables and output (39).

### 4.1 Strengths and limitations

Strengths of our study include the study population, representing one of England’s largest single centre RDCs. Moreover, we collected thorough and reliable patient-reported outcome data, in addition to comprehensive sociodemographic and clinical data. Nevertheless, our study has some limitations. Our questionnaires solely assessed patients’ anxiety and depression symptoms within two weeks prior to their initial RDC consultation. Additionally, it is crucial to scrutinize the reliability of tools like PHQ-8 and GAD-7 in measuring cancer-related distress. The conceptualization of anxiety in these measures may inadvertently pathologize normal responses to perceived threats, potentially inflating scores depending on when they are administered and failing to contextualize genuine distress. Thus, the imperative lies in developing and employing better-validated measures tailored to individuals undergoing cancer investigations, ensuring accurate assessments for the necessity of psychological interventions. Moreover, it is essential to acknowledge that the use of IMPARTS as a screening tool may overlook types of psychological distress and social problems beyond anxiety and depression, such as bereavement, eating disorders, social isolation, and other problems. A comparable unpublished clinical audit from another London RDC, with integrated psychological services, successfully identified additional unaddressed psychological needs, including addiction and alcohol use, trauma and stress, and bereavement, alongside depressive and anxiety disorders. This was achieved through targeted psychological input and the utilization of additional diagnostic tools (e.g., Short Warwick-Edinburgh Mental Well Being Scale). Therefore, additional screening tools and patient-reported outcome measures may provide a more holistic view of our patients’ psychological needs. For instance, the inclusion of the CompACT questionnaire, designed to measure the ability to adapt to situational demands in the pursuit of longer-term goals based on Acceptance and Commitment Therapy (ACT), proves particularly relevant in our patient population. This approach normalizes the human experience of fluctuations in mood and anxiety, facilitating a more meaningful identification of individuals grappling with psychological challenges during the diagnostic process. Additionally, there may be non-response bias since not all GSTT RDC attendees completed the questionnaires. **Supplementary Table 1** reveals variations in response rates, where a higher number of patients within the older age groups did not respond to the questionnaires. This may be linked with the electronic delivery of our questionnaires. It is important to note that the response rate is also likely correlated with the non-respondents unobserved health status, and therefore the likelihood of being ultimately diagnosed with cancer. Another notable limitation is the absence of follow-up data or questionnaires beyond the initial consultations. This limits our ability to discern whether patients’ anxiety or depression was a persistent issue. Lastly, another limitation of our study is the lack of detailed data on specific mental health conditions among patients with previous mental health histories. Although we observed a significant coefficient for previous mental health as a predictor variable, we are unable to fully grasp how specific mental health conditions affect the risk of severe anxiety and depression.

## 5 Conclusion

In conclusion, our study reveals a high prevalence of severe anxiety and severe depression among patients attending GSTT RDC. Fatigue, weight loss, progressive pain, and symptoms lasting more than a month emerged as combined risk factors for these psychological comorbidities. Rapid diagnostic clinics, like GSTT RDC, offer an opportunity to alleviate distress in patients with concerning symptoms by expediting diagnostic evaluations. Given the high prevalence of anxiety and depression within our NSS population, as indicated by GAD7 and PHQ8, the use Holistic Needs Assessment would be beneficial. This key screening tool, tailored for patients on an urgent cancer pathway, aids in discerning a range of needs and determining whether observed distress warrants intervention. For individuals consistently reporting distress, an upfront mental health assessment led by a mental health specialist may prove beneficial in addressing underlying mental health conditions contributing to their distress. Future research should concentrate on evaluating patient-reported Quality of Life at different time points (pre- and post-diagnosis) to further understand the trajectory of psychological distress in this NSS population. Moreover, integrating a diverse array of screening tools will provide a more comprehensive insight into the psychological well-being of patients and help identify any unmet and supportive care needs among those presenting with NSS symptoms.

## Data availability statement

The data presented in this study are available on request from the corresponding author. The data are not publicly available due to ethical reasons.

## Ethics statement

The studies involving humans were approved by GSTT Cancer Cohort (Ethics reference number: 23/NW/0105) and Health Research Authority South Central – Oxford C (Ethics reference number: 23/SC/0109). The studies were conducted in accordance with the local legislation and institutional requirements. Written informed consent for participation was not required from the participants or the participants’ legal guardians/next of kin in accordance with the national legislation and institutional requirements.

## Author contributions

MM-I: Writing – review & editing, Writing – original draft, Methodology, Investigation, Formal analysis, Data curation, Conceptualization. BR: Writing – review & editing, Supervision, Methodology. SM: Writing – review & editing, Data curation. LF: Writing – review & editing, Methodology. CLM: Writing – review & editing, Methodology, Data curation. FB: Writing – review & editing, Data curation. JM: Writing – review & editing, Data curation. LS: Writing – review & editing, Conceptualization. CM: Writing – review & editing, Data curation. CC: Writing – review & editing, Data curation. DB: Writing – review & editing, Data curation. GL: Writing – review & editing, Methodology, Data curation. AS: Writing – review & editing, Methodology. JO: Writing – review & editing, Methodology. AH: Writing – review & editing, Methodology, Funding acquisition. CR: Writing – review & editing. GJ: Writing – review & editing. SS: Conceptualization, Writing – review & editing. AS: Writing – review & editing, Methodology. MH: Writing – review & editing, Supervision, Methodology, Conceptualization. SD: Writing – review & editing, Supervision, Methodology, Data curation, Conceptualization.
