# Supplementary material for: Anxiety and depression in patients with non-site-specific cancer symptoms: data from a rapid diagnostic clinic
Source: Front Oncol. 2024 Jun 3;14:1358888. doi: 10.3389/fonc.2024.1358888 (PMC11180766; doi:10.3389/fonc.2024.1358888)
Supplement: Supplementary file 1 [file Table_1.docx]

Supplementary Table 1. Patient characteristics by IMPARTS completion status.

|  | **Completed** | **Not completed** |
| --- | --- | --- |
| **Total number of individuals** | 1734 (55%) | 1428 (45%) |
| **Sex** |  |  |
| **Male** | 667 (38.5%) | 592 (41.5%) |
| **Female** | 1067 (61.5%) | 836 (58.5%) |
| **Age at diagnosis** |  |  |
| **<25** | 30 (1.7%) | 21 (1.5%) |
| **25-50** | 443 (25.6%) | 261 (18.3%) |
| **50-75** | 998 (57.6%) | 757 (53%) |
| **>75** | 263 (15.2%) | 389 (27.2%) |
| **Index of Multiple Deprivation** |  |  |
| **Low income (1-3)** | 640 (36.9%) | 573 (40.1%) |
| **Middle income (4-7)** | 764 (44.1%) | 638 (44.7%) |
| **High income (8-10)** | 330 (19%) | 210 (14.7%) |
| **Ethnicity** |  |  |
| **White** | 922 (53.2%) | 566 (39.6%) |
| **Black** | 334 (19.3%) | 267 (18.7%) |
| **Other** | 200 (11.5%) | 148 (10.4%) |
| **Unknown** | 278 (16%) | 447 (31.3%) |
| **Smoking status** |  |  |
| **Never** | 778 (44.9%) | 414 (29%) |
| **Current** | 404 (23.3%) | 169 (11.8%) |
| **Former** | 313 (18.1%) | 205 (14.4%) |
| **Unknown** | 239 (13.7%) | 640 (44.8) |
| **Comorbidities** |  |  |
| **0** | 57 (3.3%) | 86 (6%) |
| **1** | 202 (11.7%) | 93 (6.5%) |
| **2+** | 1108 (63.9%) | 577 (40.4%) |
| **Unknown** | 367 (21.1%) | 672 (47.1%) |
| **Previous mental health illness** |  |  |
| **Yes** | 262 (15%) | 130 (9.1%) |
| **No** | 1211 (70%) | 679 (47.6%) |
| **Unknown** | 261 (15%) | 619 (43.4%) |
| **Performance status** |  |  |
| **0** | 960 (55.4%) | 351 (24.6%) |
| **1-2** | 492 (28.4%) | 409 (28.6%) |
| **3-4** | 41 (2.4%) | 56 (3.9%) |
| **Unknown** | 241 (13.8%) | 612 (42.9%) |
| **Symptom duration** |  |  |
| **<1 month** | 157 (9.1%) | 9 (0.6%) |
| **1-3 months** | 261 (15.1%) | 162 (11.3%) |
| **3-6 months** | 384 (22.2%) | 236 (16.5%) |
| **>6 months** | 600 (34.6%) | 366 (25.6%) |
| **Unknown** | 332 (19%) | 655 (46%) |
| **Weight loss** |  |  |
| **Yes** | 909 (52.4%) | 551 (38.6%) |
| **No** | 644 (37.6%) | 305 (21.4%) |
| **Unknown** | 181 (10%) | 572 (40.1%) |
| **Fatigue** |  |  |
| **Yes** | 565 (32.6%) | 301 (21.1%) |
| **No** | 974 (56.9%) | 545 (38.1%) |
| **Unknown** | 195 (10.5%) | 582 (40.8%) |
| **Vague abdominal pain** |  |  |
| **Yes** | 496 (28.6%) | 238 (16.7%) |
| **No** | 1058 (62%) | 617 (43.2%) |
| **Unknown** | 180 (9.4%) | 573 (40.1%) |
| **Progressive pain** |  |  |
| **Yes** | 567 (32.7%) | 267 (18.7%) |
| **No** | 990 (57.8%) | 589 (41.3%) |
| **Unknown** | 177 (9.5%) | 572 (40%) |

Supplementary Table 2. Stepwise regression model.

|  | **Severe anxiety** | | | **Severe Depression** | | |
| --- | --- | --- | --- | --- | --- | --- |
|  | **OR** | **95% CI** | | **OR** | **95% CI** | |
|  |  | **Lower** | **Upper** |  | **Lower** | **Upper** |
| **Socioeconomic status** |  |  |  |  |  |  |
| **Low** | 1.00 | Ref |  | 1.00 | Ref |  |
| **Middle** | 0.64 | 0.47 | 0.87 | 0.58 | 0.40 | 0.84 |
| **High** | 0.42 | 0.26 | 0.66 | 0.43 | 0.25 | 0.74 |
| **Weight loss** |  |  |  |  |  |  |
| **No** | 1.00 | Ref |  | 1.00 | Ref |  |
| **Yes** | 1.93 | 1.41 | 2.64 | 1.67 | 1.15 | 2.42 |
| **Fatigue** |  |  |  |  |  |  |
| **No** | 1.00 | Ref |  | 1.00 | Ref |  |
| **Yes** | 1.38 | 1.03 | 1.85 | 2.16 | 1.53 | 3.06 |
| **Progressive pain** |  |  |  |  |  |  |
| **No** | 1.00 | Ref |  | 1.00 | Ref |  |
| **Yes** | 1.80 | 1.34 | 2.43 | 1.58 | 1.10 | 2.25 |
| **Symptom duration** |  |  |  |  |  |  |
| **<1 month** | 1.00 | Ref |  | 1.00 | Ref |  |
| **1-3 months** | 1.94 | 1.07 | 3.52 | 1.43 | 0.70 | 2.92 |
| **3-6 months** | 1.87 | 1.06 | 3.32 | 1.89 | 0.97 | 3.66 |
| **>6 months** | 2.54 | 1.48 | 4.38 | 2.24 | 1.19 | 4.22 |
| **Previous MH illness** |  |  |  |  |  |  |
| **No** | 1.00 | Ref |  | 1.00 | Ref |  |
| **Yes** | 3.36 | 2.43 | 4.63 | 4.17 | 2.88 | 6.02 |

Supplementary Table 3. Variable importance percentages (Random Forest)

| ***Mental health condition, variable*** | ***Importance, %*** |
| --- | --- |
| **Anxiety** | |
| Number of comorbidities | 16% |
| Symptom duration | 11% |
| Performance status | 11% |
| Ethnicity | 10% |
| Age | 9% |
| Smoking | 9% |
| Socioeconomic status | 8% |
| Abdominal pain | 6% |
| Previous mental health illness | 6% |
| Fatigue | 5% |
| Progressive pain | 5% |
| Weight loss | 5% |
| **Depression** | |
| Number of comorbidities | 16% |
| Symptom duration | 11% |
| Ethnicity | 10% |
| Performance Status | 10% |
| Age | 9% |
| Smoking | 9% |
| Socioeconomic status | 8% |
| Abdominal pain | 6% |
| Weight loss | 5% |
| Previous mental health illness | 5% |
| Progressive pain | 5% |
| Fatigue | 5% |

Supplementary table 4. Final diagnosis by severe anxiety and severe depression status.

|  | Severe anxiety | | Severe depression | |
| --- | --- | --- | --- | --- |
|  | No | Yes | No | Yes |
| **Cancer** | 68 (4.6%) | 16 (6.7%) | 73 (4.6%) | 11 (6.8%) |
| **Serious benign condition** | 100 (6.7%) | 15 (6.3%) | 103 (6.5%) | 12 (7.4%) |
| **Non serious benign condition** | 458 (30.6%) | 93 (38.7%) | 492 (31.3%) | 59 (36.4%) |
| **None/Other** | 868 (58.1%) | 116 (48.3%) | 904 (57.5%) | 80 (49.4%) |
